# The role of cytokines in pediatric hematologic malignancies: mechanisms of tumor progression and therapeutic implications – a narrative review

**DOI:** 10.1097/MS9.0000000000003295

**Published:** 2025-05-21

**Authors:** Emmanuel Ifeanyi Obeagu

**Affiliations:** Department of Biomedical and Laboratory Science, Africa University, Mutare, Zimbabwe.

**Keywords:** cytokines, immune modulation, pediatric hematologic malignancies, therapeutic targeting, tumor microenvironment

## Abstract

Cytokines play a pivotal role in the progression and pathogenesis of pediatric hematologic malignancies, such as leukemia and lymphoma, by orchestrating the tumor microenvironment (TME). These soluble signaling molecules regulate key processes, including immune modulation, tumor cell proliferation, angiogenesis, and resistance to therapy. Elevated levels of pro-inflammatory cytokines such as interleukin-6 (IL-6) and tumor necrosis factor-alpha (TNF-α) promote survival pathways, chemoresistance, and immune evasion, while anti-inflammatory cytokines like IL-10 further suppress effective anti-tumor immunity. Targeting cytokine signaling pathways has emerged as a promising therapeutic strategy. Advances in monoclonal antibody therapies, signal transduction inhibitors, and cytokine-neutralizing agents aim to disrupt the pro-tumorigenic effects of cytokines within the TME. Additionally, integrating cytokine modulation with immune checkpoint inhibitors and adoptive cell therapy enhances anti-tumor immune responses. These approaches are complemented by novel therapeutics designed to mitigate resistance to conventional treatments, such as chemotherapies, which are often driven by persistent cytokine-mediated signaling.

## Introduction

Pediatric hematologic malignancies, including acute lymphoblastic leukemia (ALL), acute myeloid leukemia (AML), and non-Hodgkin lymphoma, are among the most common childhood cancers, posing significant challenges to global healthcare systems. Despite advancements in diagnostic tools and therapeutic protocols, these malignancies remain major contributors to pediatric morbidity and mortality. A key factor complicating their management is the intricate tumor microenvironment (TME), a dynamic ecosystem that influences tumor progression, immune response, and therapeutic resistance. Central to this environment is the role of cytokines, which act as critical mediators of intercellular communication and tumor-host interactions^[1,2]^. Cytokines are small, soluble proteins secreted by immune and non-immune cells, regulating processes such as inflammation, cell proliferation, differentiation, and apoptosis. In pediatric hematologic malignancies, cytokines are not merely bystanders but active participants that shape disease progression. They facilitate crosstalk between malignant cells and surrounding stromal, immune, and endothelial cells within the TME, influencing angiogenesis, immune modulation, and chemoresistance. This dual role – both supporting anti-tumor immunity and fostering tumor growth – makes cytokines pivotal targets for understanding and treating hematologic cancers in children^[3,4]^. The tumor-promoting effects of cytokines are multifaceted. Pro-inflammatory cytokines such as interleukin-6 (IL-6) and tumor necrosis factor-alpha (TNF-α) activate signaling pathways like STAT3 and NF-κB, driving tumor proliferation and survival. Meanwhile, anti-inflammatory cytokines like IL-10 and transforming growth factor-beta (TGF-β) suppress immune surveillance, enabling immune evasion by malignant cells. These cytokines also contribute to therapy resistance by protecting cancer cells from apoptosis and by supporting a permissive TME that nurtures tumor growth^[5,6]^.

**HIGHLIGHTS**

- **Cytokine dysregulation**: Abnormal cytokine levels drive inflammation, tumor progression, and metastasis in pediatric hematologic malignancies.
- **IL-6 and IL-10**: Promote tumor growth, survival, and immune evasion.
- **TNF-α & IL-1β**: Induce tumor-promoting inflammation and angiogenesis.
- **Therapeutic Targets**: Blocking cytokine pathways (e.g., IL-6 inhibitors) improves treatment outcomes.
- **Cytokine biomarkers**: Aid diagnosis, prognosis, and monitoring therapy responses.

In pediatric malignancies, cytokine dysregulation is linked to disease severity and poor prognosis. For example, elevated levels of IL-6 have been associated with increased chemoresistance and relapse in pediatric ALL, while overexpression of TGF-β correlates with immune suppression in pediatric lymphomas. Understanding the context-specific roles of cytokines is essential for unraveling the complexities of disease progression in this vulnerable population^[7,8]^. Moreover, the bidirectional relationship between cytokines and the TME underscores the potential for therapeutic interventions. Targeting cytokine-mediated pathways has gained traction as a novel strategy to disrupt the tumor-supportive functions of the TME. Monoclonal antibodies, small-molecule inhibitors, and immune checkpoint therapies are being investigated to modulate cytokine activity. For instance, drugs targeting IL-6 signaling, such as tocilizumab, have shown promise in preclinical models, paving the way for innovative treatments^[9,10]^. The implications of cytokine research extend beyond therapeutic targeting. Cytokines also serve as biomarkers for disease progression, treatment response, and relapse risk. Their levels in serum and bone marrow microenvironments can provide valuable insights into disease biology and guide personalized treatment strategies. As such, integrating cytokine profiling into clinical practice holds potential for improving prognostication and therapeutic decision-making in pediatric hematologic malignancies^[11,12]^. Despite these advancements, challenges persist. The redundancy and pleiotropy of cytokines often complicate therapeutic targeting, as individual cytokines can exert diverse and sometimes opposing effects depending on the context. Additionally, cytokine blockade therapies carry the risk of unintended immunosuppression, which can compromise host defense mechanisms, particularly in immunocompromised pediatric patients. Addressing these challenges requires a nuanced understanding of cytokine biology and the development of strategies that selectively target pathogenic cytokine signaling without disrupting normal immune functions^[13-15]^.

## Aim

This review aims to provide a comprehensive overview of the role of cytokines in pediatric hematologic malignancies, focusing on their mechanisms of action within the TME, their contributions to tumor progression and immune evasion, and their potential as therapeutic targets.

## Rationale

Pediatric hematologic malignancies, including leukemia and lymphoma, are among the most prevalent childhood cancers, significantly contributing to morbidity and mortality worldwide. Despite advancements in treatment modalities, challenges such as therapy resistance, relapse, and long-term complications remain substantial. The tumor microenvironment (TME), a dynamic and complex network of cellular and molecular components, is increasingly recognized as a critical driver of these challenges. Central to the TME’s influence are cytokines, small signaling proteins that regulate immune responses, tumor growth, and therapeutic resistance. Cytokines not only shape the interplay between malignant and immune cells but also orchestrate key processes such as angiogenesis, immune evasion, and metastasis. In pediatric hematologic malignancies, aberrant cytokine signaling often correlates with poor clinical outcomes, highlighting their pivotal role in disease progression. Despite their importance, the potential of targeting cytokine-driven pathways as a therapeutic approach remains underexplored, particularly in pediatric populations. This review aims to bridge this gap by providing a comprehensive analysis of the role of cytokines in pediatric hematologic malignancies. It seeks to elucidate the mechanisms through which cytokines contribute to tumor progression, immune modulation, and resistance to therapy. Additionally, it explores the therapeutic implications of disrupting cytokine signaling, focusing on emerging strategies and their potential integration into existing treatment paradigms. By addressing these aspects, this review underscores the need for innovative, cytokine-based approaches to improve outcomes for children with hematologic cancers.^[1-5]^

## Review methodology

### Literature search strategy

A thorough literature search was performed using electronic databases, including **PubMed, Scopus, Web of Science**, and **Google Scholar**. The search included articles published up to December 2024. Keywords and Medical Subject Headings (MeSH) terms such as **“cytokines,” “pediatric hematologic malignancies,” “tumor microenvironment,” “immune modulation,”** and **“therapeutic targeting”** were used alone and in combination to retrieve relevant studies. Boolean operators (AND, OR) were applied to refine the search strategy.

### Inclusion and exclusion criteria

Inclusion criteria were set to identify studies that:

- Investigated the role of cytokines in pediatric hematologic malignancies, including leukemia and lymphoma.
- Discussed cytokine-related mechanisms such as immune suppression, angiogenesis, chemoresistance, and metastasis.
- Explored therapeutic interventions targeting cytokine pathways in pediatric oncology.
- Included primary research, clinical trials, and relevant reviews.

Exclusion criteria excluded studies that:

- Focused exclusively on adult populations or solid tumors.
- Lacked specific information on cytokine mechanisms or therapeutic relevance.
- Were non-English articles, commentaries, or opinion pieces.

### Cytokines in the tumor microenvironment

The tumor microenvironment (TME) in pediatric hematologic malignancies is a dynamic and highly complex network where cytokines play pivotal roles in shaping disease progression. As soluble mediators of intercellular communication, cytokines orchestrate interactions between malignant cells and their surrounding stromal, immune, and endothelial cells. Within the TME, cytokines act as both facilitators of tumor-promoting processes and modulators of immune responses, contributing to the delicate balance between tumor growth and immune surveillance. This duality underscores their significance in the progression of pediatric hematologic cancers such as acute lymphoblastic leukemia (ALL), acute myeloid leukemia (AML), and various types of lymphoma^[16,17]^. Several key cytokines are implicated in the TME of pediatric hematologic malignancies, each with unique roles in promoting tumor growth, immune modulation, and angiogenesis. For instance, IL-6 is a pro-inflammatory cytokine that activates the STAT3 signaling pathway, enhancing tumor cell proliferation, survival, and chemoresistance. Elevated IL-6 levels in pediatric ALL have been associated with poor prognosis and therapy resistance. Similarly, TNF-α, a multifunctional cytokine, exhibits a paradoxical role by inducing apoptosis in low concentrations but promoting tumor growth, inflammation, and angiogenesis at higher levels. In pediatric AML, TNF-α drives nuclear factor-kappa B (NF-κB) activation, which supports tumor survival^[18,19]^.

Conversely, immunosuppressive cytokines such as TGF-β and IL-10 foster immune evasion by inhibiting cytotoxic T-cell and natural killer (NK) cell activity. TGF-β is particularly prominent in pediatric lymphomas, where it creates an immunosuppressive TME that facilitates tumor progression. Elevated IL-10 levels in pediatric hematologic malignancies suppress effective anti-tumor immune responses and are often correlated with worse clinical outcomes. These cytokines collectively enable malignant cells to evade immune surveillance, creating an environment conducive to unchecked tumor growth^[20,21]^. In addition to their role in immune modulation, cytokines significantly influence angiogenesis within the TME. Pro-angiogenic factors such as vascular endothelial growth factor (VEGF), often regulated by IL-6 and TNF-α, promote the formation of new blood vessels, ensuring an adequate supply of nutrients and oxygen to rapidly proliferating tumor cells. This angiogenic support not only fuels tumor growth but also contributes to metastasis and therapy resistance, particularly in pediatric leukemias^[22]^. The interplay of cytokines in the TME also fosters stemness and metastatic potential in malignant cells. For example, IL-6 has been shown to induce cancer stem cell-like properties, enhancing tumor aggressiveness and recurrence. Chronic exposure to pro-inflammatory cytokines within the TME can further alter the epigenetic landscape of tumor cells, driving phenotypic plasticity and increasing their adaptability to therapeutic pressures^[23]^. These insights not only provide a framework for deciphering disease biology but also open avenues for therapeutic interventions. Targeting cytokine-driven pathways within the TME holds promise for disrupting tumor-supportive processes and enhancing the efficacy of current treatment modalities. By elucidating the roles of cytokines in the TME, researchers can develop targeted therapies that selectively modulate the tumor-promoting and immunosuppressive functions of cytokines while preserving their protective roles in immune defense. This balance is crucial for improving outcomes and quality of life in children with hematologic cancers. Future studies should focus on integrating cytokine biology into the broader understanding of the TME, aiming to develop personalized and context-specific therapeutic strategies^[24]^.

### Mechanisms of tumor progression and immune evasion

Cytokines play multifaceted roles in driving tumor progression and immune evasion in pediatric hematologic malignancies. Their signaling pathways intricately influence tumor growth, angiogenesis, chemoresistance, and the suppression of anti-tumor immunity, creating a permissive environment for malignancy. The mechanisms involved are diverse and interdependent, with cytokines orchestrating cellular and molecular processes that allow malignant cells to thrive while subverting host immune defenses^[25]^. A primary mechanism by which cytokines facilitate tumor progression is through angiogenesis, the formation of new blood vessels that sustain tumor growth and metastasis. Cytokines such as vascular endothelial growth factor (VEGF), often upregulated by IL-6 and TNF-α, stimulate endothelial cell proliferation and migration, ensuring an adequate supply of oxygen and nutrients to rapidly proliferating tumor cells. In pediatric hematologic malignancies, this angiogenic support is critical for sustaining tumor viability, especially in hypoxic conditions. Additionally, pro-inflammatory cytokines like TNF-α further enhance angiogenesis by inducing the expression of matrix metalloproteinases (MMPs), which degrade the extracellular matrix to facilitate vascular remodeling^[26,27]^.

Another significant pathway is the induction of immune suppression within the tumor microenvironment. Immunosuppressive cytokines such as IL-10 and TGF-β dampen the activity of cytotoxic T cells and NK cells, key players in anti-tumor immunity. IL-10 reduces antigen presentation by dendritic cells and inhibits the production of pro-inflammatory cytokines, thereby blunting effective immune responses. TGF-β, on the other hand, promotes the differentiation of regulatory T cells (Tregs) and suppresses effector T-cell activity, creating an immunosuppressive milieu that allows malignant cells to evade immune detection. This immune evasion is particularly pronounced in pediatric lymphomas, where elevated TGF-β levels correlate with disease progression^[28]^. Cytokine-mediated activation of signaling pathways such as STAT3, NF-κB, and PI3K/AKT also contributes to chemoresistance in pediatric hematologic cancers. Persistent activation of these pathways by cytokines like IL-6 and TNF-α enhances the expression of anti-apoptotic proteins, such as Bcl-2 and survivin, which protect tumor cells from the cytotoxic effects of chemotherapy. Moreover, these pathways drive the production of reactive oxygen species (ROS), which can induce genetic mutations and epigenetic alterations, further promoting tumor adaptability and resistance to treatment^[29]^. Stemness and metastatic potential are additional hallmarks of cytokine-driven tumor progression. IL-6 and other pro-inflammatory cytokines induce epithelial-to-mesenchymal transition (EMT)-like processes in hematologic malignancies, enabling malignant cells to acquire stem cell-like properties and increased migratory capabilities. This plasticity not only enhances tumor aggressiveness but also facilitates the dissemination of malignant cells to distant sites, a key step in metastasis^[30]^. Finally, chronic cytokine signaling contributes to immune exhaustion, a state characterized by the dysfunction of T cells and other immune effectors. Prolonged exposure to cytokines such as interferon-gamma (IFN-γ) within the tumor microenvironment can induce the upregulation of immune checkpoint molecules like PD-1, CTLA-4, and TIM-3 on T cells, impairing their ability to mount effective anti-tumor responses. This phenomenon is particularly detrimental in pediatric hematologic malignancies, where robust immune responses are crucial for disease control and eradication^[30]^.

### Therapeutic implications

Targeting cytokine-driven pathways offers a promising avenue for the development of novel therapeutic strategies in pediatric hematologic malignancies. Given the significant role of cytokines in promoting tumor progression, immune suppression, and chemoresistance, modulating their activity can provide both direct anti-tumor effects and enhanced efficacy of existing treatments. This section explores key therapeutic approaches, including cytokine blockade, signal transduction inhibitors, immune modulation, and adoptive cell therapies^[31]^. Cytokine Blockade has emerged as a viable strategy to disrupt pro-tumor signaling within the tumor microenvironment. Monoclonal antibodies against cytokines like IL-6 (e.g., tocilizumab) and TNF-α (e.g., infliximab) have demonstrated clinical utility in other inflammatory diseases and are now being explored in hematologic cancers. These agents mitigate cytokine-driven proliferation and survival pathways, potentially reducing tumor burden and enhancing sensitivity to chemotherapy. Similarly, blocking TGF-β signaling with inhibitors like galunisertib has shown promise in preclinical models, reversing immune suppression and improving cytotoxic T-cell activity in the tumor microenvironment^[32,33]^. Signal Transduction Inhibitors target downstream pathways activated by cytokines, offering an alternative approach to counteracting their tumor-promoting effects. Small molecule inhibitors of STAT3, NF-κB, and PI3K/AKT signaling have demonstrated efficacy in preclinical studies, inhibiting the proliferation of malignant cells and restoring apoptotic mechanisms. For example, STAT3 inhibitors like napabucasin reduce chemoresistance by downregulating anti-apoptotic proteins and restoring the efficacy of cytotoxic agents. Similarly, NF-κB inhibitors like bortezomib can disrupt cytokine-induced inflammation and survival signaling, sensitizing tumor cells to treatment^[34,35]^.

Immune Modulation through cytokine-targeted therapies is another promising strategy to enhance anti-tumor immunity. Modulating the levels of immunosuppressive cytokines such as IL-10 and TGF-β can improve the efficacy of immune checkpoint inhibitors, such as anti-PD-1 and anti-CTLA-4 antibodies. These combinations can restore T-cell functionality and promote sustained immune-mediated tumor eradication. Furthermore, recombinant cytokines like IL-2 and IL-15, designed to boost the proliferation and activation of effector T cells and NK cells, are being tested in clinical trials to counteract the immunosuppressive microenvironment in pediatric cancers^[36,37]^. Adoptive Cell Therapies, including chimeric antigen receptor (CAR) T-cell therapy, are being enhanced by cytokine-focused interventions. Genetic engineering of CAR T-cells to resist immunosuppressive cytokines like TGF-β has shown potential in overcoming immune evasion by malignant cells. Additionally, cytokine-induced memory-like NK cells, pre-activated with IL-12, IL-15, and IL-18, are emerging as a novel adoptive cell therapy with enhanced anti-tumor activity and persistence. These strategies leverage cytokine biology to optimize cellular therapies and improve their therapeutic index in pediatric hematologic malignancies^[38]^. Another innovative approach involves the use of cytokine-neutralizing agents to reduce the side effects of systemic inflammation caused by cytokine release syndromes (CRS) during immune-based therapies. For example, tocilizumab is already used to mitigate IL-6-mediated CRS in patients receiving CAR T-cell therapy, ensuring the safety and tolerability of these advanced treatments^[39]^. Despite these advances, several challenges remain in translating cytokine-targeted therapies into routine clinical practice. The redundancy and pleiotropy of cytokine networks can limit the effectiveness of single-agent therapies, necessitating the development of combination strategies to achieve durable responses. Additionally, identifying biomarkers to predict therapeutic response and monitor treatment efficacy is critical for personalizing cytokine-based interventions^[40]^.

## Conclusion

Cytokines play a dual role in pediatric hematologic malignancies, acting as critical mediators of both tumor progression and immune regulation. These small signaling proteins influence various aspects of the tumor microenvironment (TME), including angiogenesis, immune suppression, chemoresistance, and metastasis. The therapeutic targeting of cytokine pathways represents a promising frontier in pediatric oncology. Advances in cytokine blockade, signal transduction inhibitors, immune modulation, and adoptive cell therapies highlight the potential to counteract cytokine-driven tumor progression while enhancing the efficacy of existing treatments. By addressing the immunosuppressive effects of cytokines, these strategies can restore anti-tumor immune responses and improve treatment outcomes. However, challenges such as cytokine redundancy, therapy resistance, and the identification of predictive biomarkers must be addressed to ensure the success of these interventions.

## Data Availability

Not applicable as this a review.

## Research registration unique identifying number (UIN)

Not applicable as this a review.
